# NeoSplice: a bioinformatics method for prediction of splice variant neoantigens

**DOI:** 10.1093/bioadv/vbac032

**Published:** 2022-05-06

**Authors:** Shengjie Chai, Christof C Smith, Tavleen K Kochar, Sally A Hunsucker, Wolfgang Beck, Kelly S Olsen, Steven Vensko, Gary L Glish, Paul M Armistead, Jan F Prins, Benjamin G Vincent

**Affiliations:** 1 Lineberger Comprehensive Cancer Center, University of North Carolina at Chapel Hill, Chapel Hill, NC, 27514, USA; 2 Curriculum in Bioinformatics and Computational Biology, UNC School of Medicine, Chapel Hill, NC, 27599, USA; 3 Department of Microbiology and Immunology, UNC School of Medicine, Chapel Hill, NC, 27599, USA; 4 Department of Chemistry, University of North Carolina, Chapel Hill, NC, 27599, USA; 5 Division of Hematology, Department of Medicine, UNC School of Medicine, Chapel Hill, NC, 27599, USA; 6 Department of Computer Science, University of North Carolina, Chapel Hill, NC, 27599, USA; 7 Computational Medicine Program, UNC School of Medicine, Chapel Hill, NC, 27599, USA

## Abstract

**Motivation:**

Splice variant neoantigens are a potential source of tumor-specific antigen (TSA) that are shared between patients in a variety of cancers, including acute myeloid leukemia. Current tools for genomic prediction of splice variant neoantigens demonstrate promise. However, many tools have not been well validated with simulated and/or wet lab approaches, with no studies published that have presented a targeted immunopeptidome mass spectrometry approach designed specifically for identification of predicted splice variant neoantigens.

**Results:**

In this study, we describe NeoSplice, a novel computational method for splice variant neoantigen prediction based on (i) prediction of tumor-specific k-mers from RNA-seq data, (ii) alignment of differentially expressed k-mers to the splice graph and (iii) inference of the variant transcript with MHC binding prediction. NeoSplice demonstrates high sensitivity and precision (>80% on average across all splice variant classes) through *in silico* simulated RNA-seq data. Through mass spectrometry analysis of the immunopeptidome of the K562.A2 cell line compared against a synthetic peptide reference of predicted splice variant neoantigens, we validated 4 of 37 predicted antigens corresponding to 3 of 17 unique splice junctions. Lastly, we provide a comparison of NeoSplice against other splice variant prediction tools described in the literature. NeoSplice provides a well-validated platform for prediction of TSA vaccine targets for future cancer antigen vaccine studies to evaluate the clinical efficacy of splice variant neoantigens.

**Availability and implementation:**

https://github.com/Benjamin-Vincent-Lab/NeoSplice

**Supplementary information:**

Supplementary data are available at *Bioinformatics Advances* online.

## 1 Introduction

Recent advances in immunotherapy drugs, such as CTLA-4 and PD-1 blocking antibodies (immune checkpoint inhibitors), have highlighted the capacity of induced anti-tumor immune responses to yield improved survival for cancer patients (Hellmann *et al.*, 2018; Hodi *et al.*, 2010; Larkin *et al.*, 2019). In parallel with the development of immune checkpoint inhibitors which broadly reverse effector T-cell suppression, there has also been development of therapeutic vaccines that selectively stimulate anti-tumor T cells to expand and kill tumor cells (Ito *et al.*, 2015; Keskin *et al.*, 2019; Ott *et al.*, 2017; Sahin *et al.*, 2017; Schreiber *et al.*, 2011; Schumacher and Schreiber, 2015). Tumor-specific peptide epitopes that are generated from mutated genes and presented on cell surface MHC molecules, known as neoantigens, are attractive targets for therapeutic vaccination given the lack of central tolerance and corresponding presence of endogenous T cells that recognize them (Schumacher and Schreiber, 2015). Recent studies have shown that neoantigen targeted therapy can yield improved anti-tumor immune responses in patients with advanced and metastatic tumors (Ott *et al.*, 2017; Sahin *et al.*, 2017).

The vast majority of neoantigens in tumors are patient-specific rather than shared between patients. It is therefore essential for neoantigens to be predicted for each patient in a genome-wide fashion for use in therapeutic vaccination (Schumacher and Schreiber, 2015). Currently, most available neoantigen prediction methods focus on predicting neoantigens derived from single-nucleotide variant (SNV)-derived missense mutations or insertion/deletion (indel) mutations (Smith *et al.*, 2019). Typical neoantigen prediction pipelines involve whole-exome sequencing variant calling between tumor and matched-normal samples, RNA sequencing (RNA-seq) quantification of highly expressed tumor-specific mutations and prioritizing mutation derived neo-epitopes using (among other methods) predicted binding affinities to MHC molecules expressed by the patient (Hundal *et al.*, 2016; Kardos *et al.*, 2016; Kim *et al.*, 2018; Ott *et al.*, 2017; Rajasagi *et al.*, 2014; Sahin *et al.*, 2017; Smith *et al.*, 2019; 2019; Turajlic *et al.*, 2017; Yarchoan *et al.*, 2017).

While techniques to identify and apply neoantigens for therapeutic vaccines have shown promise in several cancer types, their application may be limited in cancers that contain few somatic mutations. This has led to the investigation of alternative tumor-specific antigens (TSAs) for application in targeted therapies, including antigens derived from gene fusions, mutational frameshifts, endogenous retroviral and viral genes and splice variant sources (Smith *et al.*, 2019). An example of a low mutation tumor is acute myeloid leukemia (AML), where somatic mutation rates are notably lower in comparison to other cancer types (Cancer Genome Atlas Research Network, 2013). However, mutations in spliceosomal genes and genome-wide aberrant splicing events are common in patients with AML (de Necochea-Campion, 2016; Lee *et al.*, 2016). To extend therapeutic neoantigen targeting to AML and other low mutation tumors with significant alternative splicing, it will be critical to identify splice variant neoantigens. Yet, the prediction of neoantigens from tumor-specific splice variations presents significant computational challenges. Recently, two splice variant neoantigen prediction methods were reported with analysis of the Cancer Genome Atlas (TCGA) pan-cancer dataset (Jayasinghe *et al.*, 2018; Kahles *et al.*, 2018). However, these tools were not designed to predict the full length splice variant transcript, simply the alternative splice junctions themselves. As such, predicted variant junctions are limited in their capacity to predict transcriptional start sites and translational reading frames. Other tools for prediction of splice variant junctions (Brooks *et al.*, 2014; Kahles *et al.*, 2016; Rogers *et al.*, 2012; Shen *et al.*, 2014; Zhang *et al.*, 2020), splice variant neoantigens (Smart *et al.*, 2018; Zhang *et al.*, 2020) and somatic mutations that predict for alternative splicing (Jayasinghe *et al.*, 2018; Mort *et al.*, 2014) have also been reported in the literature. However, many tools lack robust evaluation of performance characteristics using *in silico* simulated data (Smart *et al.*, 2018; Zhang *et al.*, 2020). More importantly, few tools provide proteomic validation of predicted splice variant neoantigens (Smart *et al.*, 2018; Zhang *et al.*, 2020), with no tool providing immunopeptidome validation against a synthetic peptide reference of predicted antigens. As such, the true biological performance of these tools cannot be evaluated. Altogether, the paucity of robust splice variant neoantigen prediction tools has limited the capacity to study the therapeutic potential of these semi-public TSAs, with no study to date demonstrating concrete evidence in favor of anti-tumor immunity generated by splice variant neoantigens. The need for robust TSA prediction tools is underscored by recent studies providing growing evidence supporting the importance of splice variant neoantigens and other alternative TSA in antitumor immunity (Ehx *et al.*, 2021).

We developed NeoSplice to address the challenge of accurate computational prediction of splice variant neoantigens. Using a Burrows Wheeler Transform (BWT) based algorithm to identify tumor-specific k-mers and a splice graph to determine whether such a k-mer represents a tumor-specific splice junction in a coding region and its corresponding amino-acid sequence, NeoSplice allows for specific and comprehensive prediction of splice variant neoantigens using tumor and matched-normal RNA-seq data, without requiring matched DNA sequencing data. In this report, we describe the NeoSplice method, validating performance characteristics using *in silico* simulated RNA-seq data. We then apply NeoSplice to TCGA LAML dataset, demonstrating significantly greater sharing of splice variant-derived neoantigens as compared to SNV-derived neoantigens. Lastly, we biologically validate NeoSplice’s predictions in the K562.A2 AML cell line, using mass spectrometry targeted identification of predicted splice variant neoantigens from the immunopeptidome.

## 2 Methods

### 2.1 The NeoSplice method

**Fig. 1.**
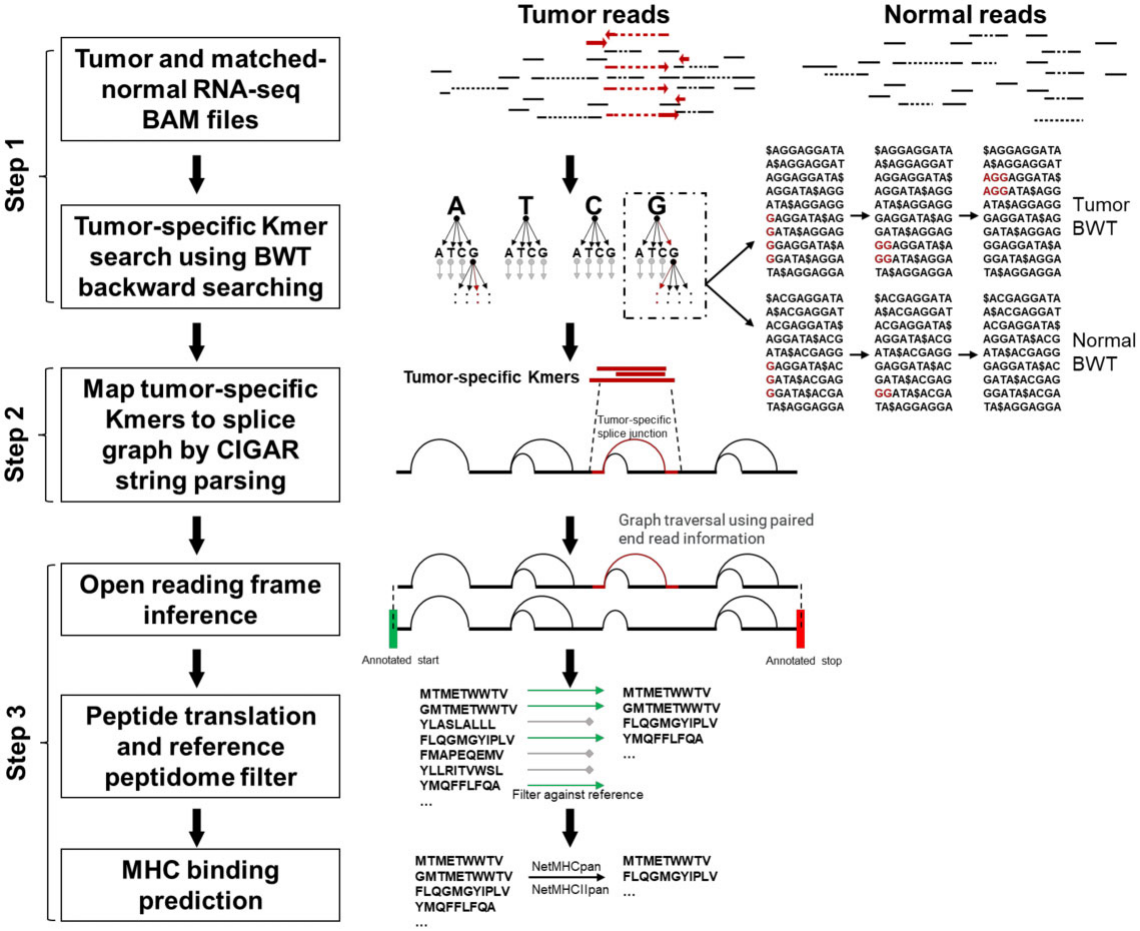
Overview of the NeoSplice method. BAM files from tumor and matched-normal samples generated from a splice-aware aligner are input into the NeoSplice algorithm. Step 1: The multi-string BWT tool based on a variant of the Burrows Wheeler transform (BWT) builds the multi-string BWT data structure for tumor and normal RNA-seq bam files. Step 2: The splice graphs are constructed from the tumor RNA-seq bam files. A depth-first search process operating in lockstep on the tumor and normal BWT data structures identifies all tumor-specific k-mers. The tumor-specific k-mers are mapped onto the splice graphs using CIGAR strings. Step 3: Graph traversal infers the tumor-specific splice junction containing partial transcript isoforms within an open reading frame by taking advantage of using paired-end read information and annotated transcript information. Sequences spanning tumor-specific splice junctions are translated into amino acid space, filtering against a reference peptidome. Remaining peptide sequences are run through MHC binding prediction software, with predicted binders representing putative splice variant neoantigens

Multiple steps are needed to identify a novel splice that occurs specifically in tumor cell transcripts whose translation will result in a neopeptide that can be targeted by T cells (Fig. 1):

- Step 1: Tumor-specific k-mer generation. Using one RNA-seq dataset ***T*** of tumor cells and one RNA-seq dataset ***N*** of normal cells, tumor-specific k-mer sequences present abundantly in the transcriptome of the tumor cell, but not/rarely expressed in the normal cells are identified.
- Step 2: Prediction of splice variant transcripts. The splice graph ***G*** from the tumor cell RNA-seq data is built, and tumor-specific k-mers from above are mapped to novel splice variant transcripts. Gencode annotations are used to determine whether the novel splice lies within a protein coding region and infer the reading frame of the transcript.
- Step 3: Prediction of splice variant neoantigens. Novel splice junctions contained within each splice variant transcript are translated in the inferred open reading frame. MHC binding affinity prediction is performed on translated peptide sequences to determine which novel regions may yield a neopeptide.

Further breakdown and details of each step is described in Supplementary methods.

### 2.2 Generation of simulated reads and performance characterization

Protein coding regions of annotated hg38 transcripts were modified to generate tumor-specific splice junctions resulting in novel transcripts. One tumor-specific splice junction type was selected from exon skipping splice junction type, partial exon loss splice junction type or partial intron gain splice junction type for data simulation (Supplementary Fig. S1). These events cover the majority of splicing abnormalities observed in AML. For each chromosome, five protein coding genes with greater than or equal to eight annotated transcript isoforms were randomly selected. One tumor-specific splice junction per selected gene was then simulated by randomly choosing a combination of donor splice site and acceptor splice site that does not match any splice junctions in the annotated protein coding regions of transcripts for that gene and forms a potential novel splice junction with specified splice junction type compared with reference transcripts. The selected novel splice junction was then applied to all annotated transcripts of that gene. Reference transcripts for comparison to tumor transcripts were written to a separate GTF file and used as normal data. Transcripts that cannot form the specified splice junction type by applying the novel splice junction and genes with no possible tumor-specific splice junctions were excluded from simulated tumor and normal data.

RNA-seq simulator Polyester (v1.9.754) (Frazee *et al.*, 2015) was used to simulate reads using the generated GTF file as input. One thousand 100 bp paired-end reads were simulated for each transcript in the GTF file. 20 random bootstrap tumor-normal pair datasets were simulated in order to obtain more general results. STAR 2.7.5e (Dobin *et al.*, 2013) was run using simulated reads as input to generate bam file and splice junction output SJ.out.tab. Splice junctions identified by STAR in tumor and normal with less than or equal to 10 uniquely mapping reads spanning the splice junction were filtered. Then the splice junctions in tumor that do not present in splice junctions in normal were considered as ground truth tumor-specific splice junctions. The STAR output bam file was then run through NeoSplice (T_min = 21, N_max = 3), comparing predicted versus simulated splice variants for performance calculations. ASNEO (Zhang *et al.*, 2020) was run with default parameters using STAR splice junction output SJ.out.tab as input file.

### 2.3 Generation, culture and mRNA sequencing of K562.A2 cell line

The K562 (ATCC^®^ CCL-243™) (Lozzio and Lozzio, 1975) cell line transduced with a retroviral vector to stably express HLA-A*02:01 (K562.A2) was a kind gift from Dr. Barbara Savoldo (The University of North Carolina at Chapel Hill). The cells were cultured in RPMI 1640 (Gibco, Thermo Fisher Scientific) supplemented with 10% fetal bovine serum (Hyclone Laboratories, Inc., GE Healthcare), 100 U/ml penicillin-streptomycin (Gibco, Thermo Fisher Scientific) and 2 mM l-glutamine (Gibco, Thermo Fisher Scientific). RNA was isolated from K562.A2 cells using Qiagen’s RNeasy Plus Mini Kit, with RNA eluted in RNase-free water. The sequencing library was prepared at the High Throughput Sequencing Facility (The University of North Carolina at Chapel Hill), using the KAPA Stranded mRNA-Seq Kit Illumina Platforms (Roche Sequencing), and paired-end sequencing (150 cycles/end) was performed on an Illumina HiSeq4000.

### 2.4 Isolation and mRNA sequencing of CD34+ cells

Four leukapheresis products from GCSF mobilized healthy allogeneic stem cell donors that were to be discarded otherwise were thawed, and PBMCs were isolated using Ficoll-Paque gradient centrifugation. Lineage positive cells were removed using the Lineage Depletion Kit (Miltenyi), and CD34+ cells were isolated from the lineage-depleted PBMCs using the CD34 Microbead Kit, Ultrapure (Miltenyi). Total RNA was purified using the RNeasy Plus Mini Kit (Qiagen). The sequencing libraries were prepared at the High Throughput Sequencing Facility (The University of North Carolina at Chapel Hill) using the TruSeq RNA Sample Prep Kit (Illumina), and pair-end sequencing (100 cycles/end) was performed on an Illumina HiSeq2000.

### 2.5 Splice variant neoantigen prediction for K562.A2 and TCGA LAML

For the K562.A2 cell line, STAR 2.7.5e (Dobin *et al.*, 2013) and ABRA2 2.19 (Mose *et al.*, 2019) were used for RNA-seq read alignment to the GENCODE GRCh38.p13 human reference. STAR two-pass mode aligned RNA-seq bam files for TCGA tumor and adjacent normal pairs were downloaded from GDC (https://portal.gdc.cancer.gov) (*n* = 137). CD34+ hematopoietic stem cell RNA-seq data were used as reference normal data. NeoSplice was run on the K562.A2 cell line RNA-seq BAM file to predict splice variant neoantigens. In the tumor-specific k-mer searching stage, tumor thresholds of T_min = 21 and N_max = 3 were used. Tumor-specific k-mers length were restricted to be at most 90% of read length. In the splice graph generation stage, the minimum number of supporting reads for including SNVs, insertions and deletions in the splice graph were set to 10 and the *P*-value threshold for calling SNV edges was set to 0.000005. Tumor-specific k-mer graph paths that contain splice junctions found by RNA-seq alignment to be supported by at least 20 reads in tumor RNA-seq data but supported by <3 reads in normal RNA-seq data were considered. Tumor-specific k-mer graph paths supported by <15 tumor-specific k-mer-including reads were filtered. Peptides that were also present in GRCh38.p13 reference peptidome generated by translating reference protein coding transcripts in the Gencode GFF3 file (gencode.v35.annotation.gff3) were filtered. Mutant peptides were run through netMHCpan4.0 (Jurtz *et al.*, 2017) (peptide length 8-11mer) for HLA binding prediction, using HLA-A*02:01 for K562.A2 and OptiType (Szolek *et al.*, 2014) calls available from GDC. Predicted neoantigens were defined as peptides with predicted binding affinity <500 nM (Rajasagi *et al.*, 2014).

### 2.6 SNV neoantigen prediction for TCGA LAML

SNV-derived neoantigens for TCGA LAML samples were derived using GDC MAF files, using methods adopted from Thorsson *et al.* (2019). Somatic nonsynonymous coding SNVs were extracted from the MC3 variant file (mc3.v0.2.8.PUBLIC.maf) with the following filters: FILTER in ‘PASS’, ‘wga’, ‘native_wga_mix’; NCALLERS > 1; Variant_Classification = ‘Missense_Mutation’; and Variant_Type = ‘SNP’. For each SNV, the Ensembl protein reference sequence was obtained, and the minimal peptide encompassing the mutation site plus 10 amino acids up and downstream of the mutation site was extracted (21 aa long peptide). If the mutation occurred within 10 amino acids of the N- or C-terminal end of the protein, all available sequence between the mutation and start/end of the protein was taken, resulting in a minimal peptide shorter than 21aa. The variant position within the minimal peptide was recorded, and the mutation was applied to the minimal peptide, resulting in a mutant minimal peptide. Mutant peptides were run through netMHCpan4.0 (Jurtz *et al.*, 2017) (peptide length 8-11mer) for HLA binding prediction, using OptiType (Szolek *et al.*, 2014) HLA calls available from GDC. Predicted neoantigens were defined as peptides with predicted binding affinity <500 nM.

### 2.7 HLA peptide isolation

Peptide epitopes presented by HLA-A*02:01 were isolated using a modified version of the protocol from Park *et al.* (2017). Five hundred million K562.A2 cells were washed 3 times in Dulbecco’s phosphate buffered saline (DPBS; Gibco, Thermo Fisher Scientific) and lysed in 50 ml of ice-cold IP-lysis buffer: 1x DPBS, 1% Triton X-100 (Sigma-Aldrich), 5 mM EDTA (Thermo Fisher Scientific), 1× Halt Protease Inhibitor (Thermo Fisher Scientific), 1 mg/ml leupeptin (R&D Systems) and 1 mM PMSF (phenylmethylsulfonyl fluoride; Sigma-Aldrich). The lysate was incubated on ice for an hour and vortexed every 15 min, then cleared by centrifugation for 30 min at 3000×*g* at 4°C. The protein concentration of the supernatant was measured using the Coomassie Plus Bradford Protein Assay (Thermo Fisher Scientific). The cleared lysate was incubated on a rotator overnight at 4°C with 2 g of HLA-A*02-specific BB7.2 Ab (BioLegend) per mg of lysate protein, followed by a 4 hour incubation with Protein A/G Ultralink Resin (Thermo Fisher Scientific). After washing 3 times with DPBS, the resin was transferred to a 2 mL centrifuge column (Pierce, Thermo Fisher Scientific). HLA-A*02:01-peptide complexes were eluted by gravity flow using ice-cold 0.1 N acetic acid (Thermo Fisher Scientific) in 5 fractions, each equal to a column volume. The fraction containing eluted proteins was determined using the Bradford Assay, and glacial acetic acid was added to 10% of the final volume of this fraction to separate the peptides from HLA-A*02:01. The sample was filtered through a 10 kDa regenerated cellulose filter (Amicon, EMD Millipore) to separate the peptides from larger proteins and stored at −80°C until analysis.

### 2.8 Mass spectrometry analysis of the immunopeptidome

Electrospray ionization coupled to differential ion mobility spectrometry-mass spectrometry (ESI-DIMS-MS) was used at a dispersion field (ED) of 24 kV/cm and a compensation voltage of 2.0 V. The mass spectrometer was tuned to optimize transmision of singly charged species that range from m/z 864 to m/z 1348. The MS/MS spectra acquired from collision-induced dissociation (CID) of the singly charged species from the cell sample were compared and matched with the MS/MS spectra obtained from synthetic peptide standards (Flashpure Custom Peptide Array, New England Peptide, Inc.), predicted from NeoSplice analysis of the K562.A2 cell line. A library of the predicted peptide standards was developed using the instrument vendor software (Bruker DataAnalysis LibraryEditor). The software was used to compare MS/MS spectra from real samples to a spectral library of predicted peptide standards obtained using the same instrumental conditions. The Fit score (F) indicates how well masses and intensities of the library spectrum (assumed to be pure) and acquired spectrum (contains other signals) agree, with a maximum score of 1000. An acquired MS/MS spectrum identified with an F score of >700 was selected as a positive match.

### 2.9 Availability

The NeoSplice software is available on the GitLab page: https://github.com/Benjamin-Vincent-Lab/NeoSplice. The Docker image for NeoSplice is available on: https://hub.docker.com/r/benjaminvincentlab/neosplice.

## 3 Results

### 3.1 Performance and resource utilization characteristics

**Fig. 2.**
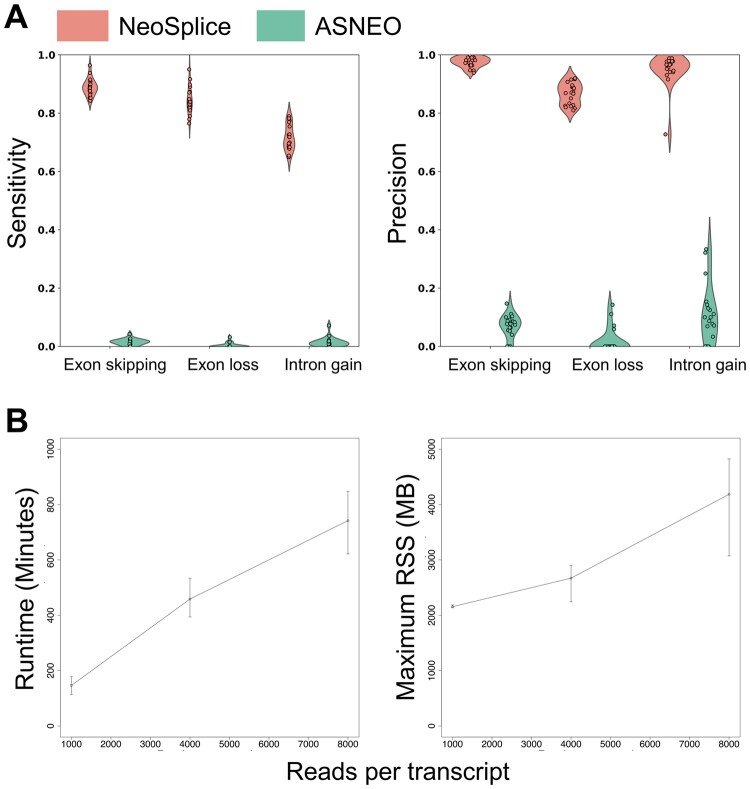
Performance and resource usage of NeoSplice on simulated data. (**A**) Sensitivity and precision for simulated splice variant transcripts, including those derived from exon skipping, exon loss, or intron gain. Values represent the mean performance across 22 chromosomes (up to 5 splice variant junctions per chromosome) for each of 20 total simulated samples. (**B**) Average runtime (left) and max resident set size (RSS; right), with error bars representing the maximum and minimum times across three simulated samples. Runtime is defined as the elapsed time and RSS is the amount of memory requested by NeoSplice from the operating system as reported by the ‘sacct’ command, as measured on an Intel Xeon ES-E5620 2.4 GHz CPU or ES-E5520 2.27 GHz CPU

The main computational challenge for prediction of splice variant neoantigens is inference of tumor-specific splice junctions contained within transcripts. To assess the performance of NeoSplice, we simulated 20 RNA-seq datasets using hg38 splice junction annotations to include novel, unannotated protein coding splice junctions for each splice junction type, including exon skipping splice junctions (∼40% of all alternative splicing events in higher eukaryotes), partial intron gain splice junctions (<5% of all alternative splicing events in vertebrate and invertebrates), and partial exon loss splice junctions (∼25% of all alternative splice events in higher eukaryotes) (Keren *et al.*, 2010). Five protein coding genes were selected for each chromosome. One tumor-specific splice junction was selected for each gene if a tumor-specific splice junction was supported; genes with no possible tumor-specific splice junctions were excluded. We then used Polyester31 to simulate 100 bp paired-end reads from the simulated transcript GTF files, with 8000 reads simulated per transcript. The median sensitivity and precision values of NeoSplice were greater than 0.8 for all types of splice junctions except intron gain sensitivity (0.71) (Fig. 2A). The performance of NeoSplice was also compared with another splice variant neoantigen prediction tool, ASNEO23, showing significantly improved median sensitivity and precision values (Fig. 2A). ASNEO relies upon an external reference for normal samples (hg19 reference (Church *et al.*, 2011) and GTEx (GTEx Consortium, 2017)), highlighting the benefits and importance for using matched normal data for accurate prediction of splice variants. Notably, ASNEO relies upon a built-in reference derived from hg19 while NeoSplice’s reference can be user-selected. As we were unable to change ASNEO’s reference, performance variation between the two tools may be attributable to both functional differences between the two algorithms as well as NeoSplice’s use of hg38.

To determine resource requirements of NeoSplice, we measured the running time and memory consumption using simulated transcript sets containing 1000, 4000 and 8000 reads per transcript. Runtime and memory consumption (maximum resident-shared size; recorded using ‘sacct’ command) increase linearly in proportion to the number of reads simulated per transcript, as expected for NeoSplice (Fig. 2B).

### 3.2 Analysis of splice variant neoantigens in TCGA LAML

**Fig. 3.**
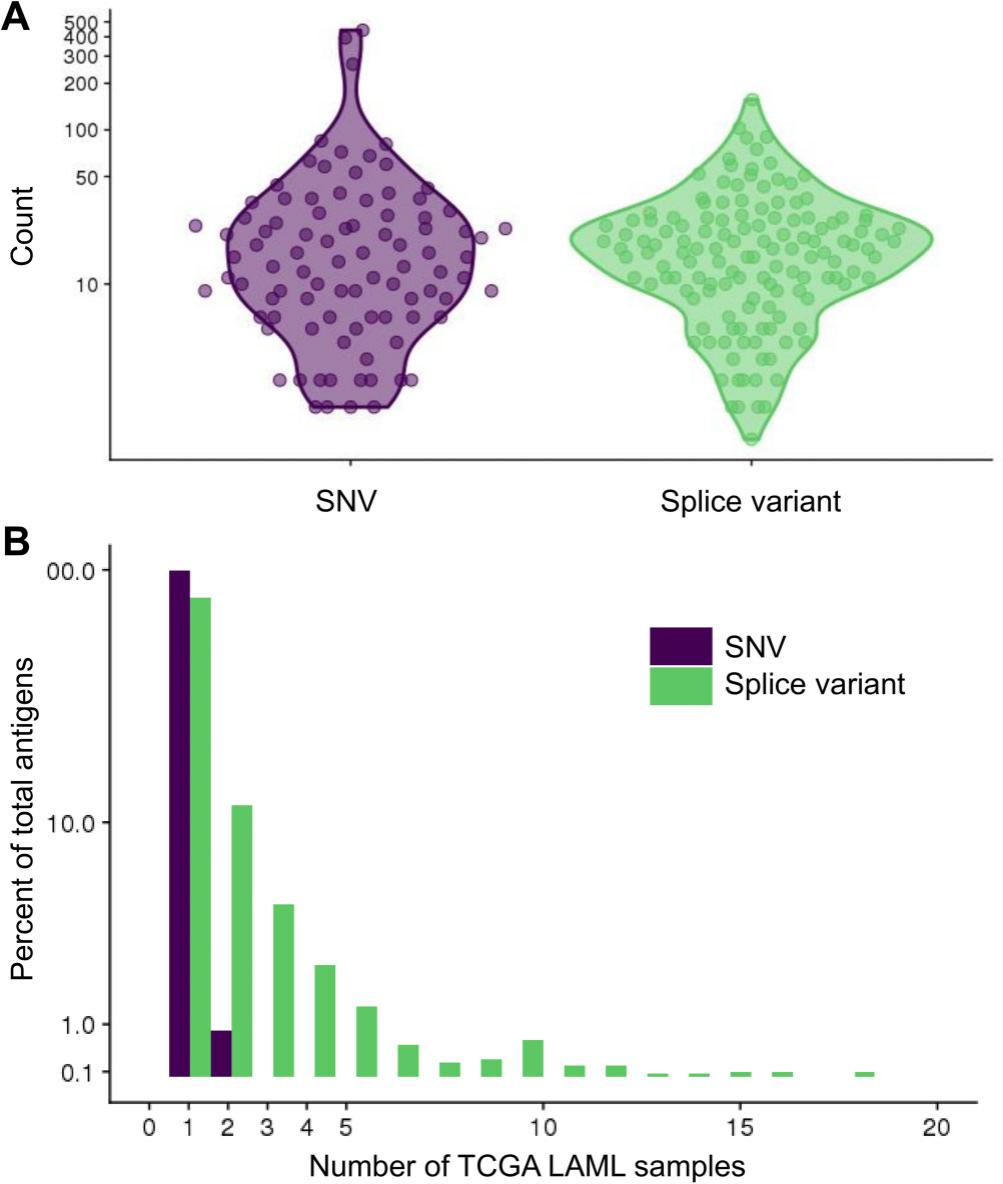
Comparison of single-nucleotide variant (SNV) and splice variant (SV) antigen calls in the TCGA LAML dataset. (**A**) Number of HLA-I SNV and SV antigen calls per TCGA LAML sample, defined as netMHCpan4.0 predicted binding affinity <500 nM. (**B**) Histogram showing proportion of public SNV or SV antigens shared among TCGA LAML samples

To elucidate the splice variant neoantigen landscape of various cancer types, we next performed NeoSplice analysis in the TCGA LAML dataset. To assess the distribution of splice variant neoantigens in different types of cancer, NeoSplice was run on TCGA LAML samples (*n* = 137) using healthy donor bone marrow-derived CD34+ hematopoietic stem cells as a reference normal. Of 19 970 total genes that showed expression, 316 demonstrated splice variants, representing 1872 unique predicted MHC binding peptides (Supplementary Fig. S2A and B). Among predicted splice variants, the derivative genes were often shared between samples, including 34 genes contained within the predicted splice variants of >5% of samples (Supplementary Fig. S2C). The most prominent of these genes was MPO, seen in ∼35% (48 of 137) of samples. The number of SNV and splice variant neoantigens were similar with TCGA LAML (Fig. 3A; median count per sample: SNV = 14, splice variant = 17; Welch *t*-test *P* = 0.18). We did not observe a significant difference in splice variant neoantigen levels between samples containing spliceosomal mutations in *U2AF1*, *SRSF2* or *SF3B1* genes (median = 15; SD 13.0) versus those without these spliceosomal mutations (median = 17; SD 22.9). While SNV neoantigens are generally patient-specific, we observed sharing of splice variant neoantigens across TCGA samples, including seven splice variant neoantigens that were observed in >10% (>13) of all TCGA LAML samples (Fig. 3B).

### 3.3 Mass spectrometry validation of NeoSplice predicted antigens

**Fig. 4.**
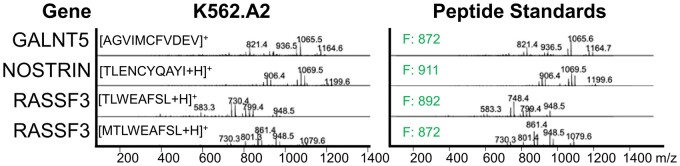
Mass spectrometric analysis of the K562.A2 cell line HLA-I immunopeptidome versus NeoSplice predicted peptides. MS spectra from K562.A2 anti-HLA-A*02-based immunoprecipitation and column purification/acid elution of MHC-bound peptides (left) alongside the matching peptide standard from NeoSplice predicted antigens from the K562-A2 cell line (right). Fit (F) score for each peptide is shown (right).

To biologically validate NeoSplice predicted splice variant neoantigens, a set of **N** neoantigen peptides predicted in the K562.A2 cell line (an AML cell line engineered to express HLA-A*02:01) were synthesized. HLA-A*02:01 binding peptides were isolated from K562.A2 cells using anti-HLA-A*02-based immunoprecipitation and column purification/acid elution of the MHC-bound peptides. Using synthesized peptides as reference for electrospray ionization coupled to differential ion mobility spectrometry-mass spectrometry (ESI-DIMS-MS), we searched for the presence of these predicted splice variant neoantigens in the K562.A2 MHC ligand pool. Comparing MS/MS spectra from K562.A2 to a spectral library of predicted peptide standards obtained using the same instrumental conditions, we identified 4/37 (10.8%) of predicted antigens from the immunopeptidome of one K562.A2 sample, which corresponded to 3/17 (17.4%) unique predicted splice junctions identified by MS (Fig. 4).

### 3.4 Comparison of NeoSplice with other methods for splice variant neoantigen prediction

Table 1 compares NeoSplice with eight other methods for prediction of splice variants. While the majority of these tools only predict novel splice junctions, NeoSplice, ASNEO and the RI neoantigen pipeline further predict the neoantigens derived from these splice junctions. MutPred Splice and MiSplice are unique in using DNA-seq data to predict for somatic variants that may disrupt normal splicing of mRNA, while remaining tools are similar to NeoSplice in predicting splice variants using RNA-seq data only.

**Table 1.** Comparison of splice variant prediction tools from the literature with NeoSplice

Splice variant antigen caller	Input	Predicts neoantigens	Splicing event identified	Required packages	*In silico* performance	Wet lab validation	Wet lab performance
ASNEO	RNA-seq	Yes	Filters reads against GTEx and hg19 reference, translating novel isoforms into proteins for antigen prediction.	Python: sj2psi R: survival, survminer, MCPcounter	Not reported	Mass spectrometry (external dataset)	2/407 peptides confirmed from 14 patient cohort
JuncBase	RNA-seq	No	(1) Identifies annotated and novel splice junctions, (2) quantifies each junction and (3) calculated for differential expression between groups.	Python 2.6+ Biopython 1.5+ Pysam R v2.14+ Rpy2 MySQL/sqlite	Sens. ∼50–80% Prec. ∼10–95% (Compared in Kahles *et al.*)	RT-PCR	16/16 splicing events confirmed
MiSplice	RNA-seq + WGS	No	Jointly analyzes WGS and RNA-Seq data, scanning the transcriptome for statistically significant non-canonical sequence junctions supported by expression evidence.	SamTools MaxEntScan	Sens. 74–97% Spec. ∼77%	Splicing reporter minigene functional assay	10/11 of splicing alterations
MutPred Splice	DNAseq	No	Uses human disease alleles for training a machine learning model to predict exonic nucleotide substitutions that disrupt pre-mRNA splicing.	None (web interface)	FPR = 7.0% Sens. 64.7% Spec. 93.0% Acc. 78.8% AUC 83.5%	RT-PCR	Amplicon changes from ATM mutation-contain vs WT cell line confirmed by RT-PCR
NeoSplice	RNA-seq	Yes	(1) Identify differentially expressed k-mers, (2) map tumor-specific k-mers to splice graph and (3) ORF inference, translation, and MHC binding prediction.	Python 2.7 MSBWT MSBWT-IS NetMHCpan 4.0 NetMHCIIpan 3.2 networkx 1.11 pyahocorasick 1.4.0 bcbio-gff 0.6.4 pyfaidx 0.5.3.1 pysam 0.14.1 biopython 1.70 scipy 1.2.0	Sens. >80% Recall >80%	Internal mass spectrometry validation against synthetic peptide reference	4/37 peptides confirmed, corresponding to 3/17 novel splice junctions
RI neoantigen pipeline	RNA-seq	Yes	(1) Pseudoaligns RNAseq reads to hg19 with exon and intron transcripts, (2) quantification, (3) KMA algorithm to identify expresed introns and (4) predict MHC binding.	Kallisto KMA suite of python and R packages POLYSOLVER NetMHCpan v3.1	Not reported	Mass spectrometry (external dataset)	Confirmed 1–2 per each of six cell line tested (Mean total splice variant neantigen load of 1515)
rMATS	RNA-seq	No	Detection of differentially expressed splice variants between two sets of RNA-seq data.	Python 2.7/3.6 BLAS, LAPACK GSL 2.5 GCC (5.4.0) Fortran 77 CMake (3.15.4)	Sens. ∼30% Prec. >95% (Compared in Kahles *et al.* for exon skipping only)	RT-PCR	32/34 exon skipping candidates confirmed
SplAdder	RNA-seq	No	(1) Integrating annotation and RNA-Seq data, (2) generating an augmented splicing graph, (3) extraction of splicing events, (4) quantifying the events, and optionally and (5) the differential analysis between samples.	LIMIX GATK STAR Samtools	Sens. ∼30–80% Prec. ∼20–90%	None	NA
SpliceGrapher	RNA-seq +/-EST	No	(1) Alignment of RNA-seq to the reference genome, (2) spliced alignment of reads that did not align in the first step, (3) initial splice graph construction, (4) assembly of exons from the ungapped short-read alignments and (5) insertion of the new exons into the splice graph using spliced alignments.	PyML 0.7.9+ matplotlib 1.1.0+ pysam 0.5+	Sens. ∼30–60% Prec. ∼20–90% (Compared in Kahles *et al.*)	None	NA

An important difference between these splice variant calling tools are the methods utilized to validate the predictions. Several tools (SplAdder, SpliceGrapher) did not report any wet lab validation studies, while the majority reported RT-PCR based strategies to confirm splice products. Only three tools included mass spectrometry validation of the translated protein products from predicted splice variant neoantigens: ASNEO, the RI neoantigen pipeline, and NeoSplice. Smart *et al.* (2018) (RI neoantigen pipeline) utilized mass spectrometry to validate predicted neoepitopes and identified 1–2 of the 5000–10 000 predicted RI neoepitopes and 0–2 of the 400–10 000 predicted somatic neoepitopes per cell line tested. Zhang *et al.* (2020) (ASNEO) confirmed 2 of 407 predicted MHC-I neoantigens by mass spectrometry. In comparison, our study identified 4 of 37 peptides by mass spectrometry, including validation of 3 of 17 unique splice junctions. In contrast to the two above studies, the mass spectrometry method described in this study used a synthetic peptide reference to compare the immunopeptidome spectra against, increasing the confidence of validated peptides. Although the percentage of confirmed antigens cannot be directly compared between our method versus the two above studies, the increased sensitivity and accuracy of having a peptide reference allowed for validation of a much higher percentage of tested splice variant neoantigens.

## 4 Discussion

In this report, we describe NeoSplice, a bioinformatics tool for prediction of splice variant neoantigens from RNA-seq data. The performance comparison results from the simulated data indicates that predictions made by NeoSplice are highly sensitive and precise. Moreover, NeoSplice is a time and space efficient algorithm. Our novel BWT-based tumor-specific k-mer searching algorithm does not need to examine all possible k-mers in tumor and normal RNA-seq data, and the BWT method requires less memory compared to hash-table based methods that usually require large memory to build an index. The splice graph building step requires time proportional to the number of reads in the data set and the splice variant peptide prediction step requires time proportional to the number of edges and nodes in the connected component that contains the tumor-specific splice junction. Moreover, NeoSplice can be extended to predict neoantigens derived from other types of mutations such as SNVs, indels, and gene fusions as identification of tumor-specific k-mers should capture each of these and the splice graph traversal will identify the transcripts that contain tumor-specific mutations.

In contrast to SNV-derived neoantigens, many splice variant neoantigens are found to be shared among different AML tumors in our analysis, suggesting the feasibility to develop an off-the-shelf splice variant neoantigen therapeutic. We expect that NeoSplice will expand the neoantigen therapeutic target space for cancer patients, possibly providing an avenue for off-the-shelf therapeutics. Currently, there are over 160 clinical trials of therapeutic neoantigen vaccines in cancer registered on ClinicalTrials.gov. Multiple companies have others, along with neoantigen-specific adoptive cellular therapy approaches, in development. Accurate identification of splice variant neoantigens will become more important as additional neoantigen-specific therapeutic platforms enter clinical trials, especially for those tumors like AML where neoantigens derived from SNVs and Indels are rare.

NeoSplice provides several improvements upon current splice variant prediction methods described in the literature. Firstly, NeoSplice is one of few tools that incorporates alternative splice prediction alongside antigen prediction. This is bolstered by the capacity of NeoSplice to predict the full length splice variant transcript upstream of novel splice junction and the partial splice variant transcript downstream of novel splice junction, with open reading frame inference to predict for the in-frame peptide. Secondly, NeoSplice performed very well with *in silico* validation, with sensitivity and precision >80% for the majority of simulated splice variant transcripts, which is significantly higher compared against a recently published splice variant prediction tool ASNEO. Importantly, NeoSplice’s superior performance may be attributable to both the algorithm itself as well as the use of the newer hg38 reference, which matched the reference used to generate the simulated dataset. This comparison ideally should be performed using an updated version of ASNEO containing the hg38 reference; however, ASNEO’s resources did not contain information on how to exchange the built-in reference. Notably, hg38 contains more comprehensive annotations for splice junctions and should ideally be used for best splice variant prediction performance. This also highlights NeoSplice’s advantage in being able to adapt newer references as they are developed. In addition to ASNEO, most other tools did not provide *in silico* validation performance, making it difficult to interpret the robustness of the tool for downstream antigen selection. Thirdly, we also report here the runtime and memory requirements for NeoSplice—an important consideration given the computationally intensive nature of predicting for alternative splice variants and limited timeframe for selection of cancer antigen targets for therapeutic vaccine development. Similarly, these characteristics are not widely reported in other tools. NeoSplice’s efficiency allows for large datasets (such as TCGA cohorts) to be analyzed, with runtimes that are compatible with timelines for cancer antigen vaccine development. Lastly, our mass spectrometry validation approach is a unique strength of this study. Unlike previous studies that also examined mass spectrometry data, this is the first study to our knowledge that generated internal mass spectrometry data and used synthetic reference peptides to compare against immunopeptidome spectra. As such, while a smaller percentage of total predicted splice variant antigens were tested, the confidence of the positive mass spectrometry hits was increased. Additionally, while we tested only 37 peptides, we were able to validate >10% of antigens comprising >17% of all unique splice variant transcripts contained in the reference set of peptides. Given the sensitivity of mass spectrometry is imperfect, we expect the true positive rate to be further increased from these estimates. Lastly, we only used mass spectrometry evaluation of a subset of peptides selected as optimal vaccine candidates. As such, our validation provides a more direct metric for likelihood of a valid vaccine candidate, as compared to previous studies which evaluated the entire pool of predicted splice variant neoantigens. We believe the validation provided in this study is the most biologically robust of any currently described method, with greatest relevance for downstream antigen-specific therapeutics. Overall, we believe these advantages make NeoSplice a powerful tool for predicting splice variant neoantigens, providing the computational framework for future studies to test the efficacy of splice variant targeted vaccine and cellular therapies.

## Funding

This work has been supported by the National Institutes of Health Clinical Center (1F30CA225136, 5R01CA201225, R37CA247676 and T32CA196589) and the University of North Carolina University Cancer Research Fund.

*Conflict of Interest:* B.G.V. has equity in GeneCentric Therapeutics.

## Data Availability Statement

The data underlying this article are available in the article and in its online supplementary material.

## Supplementary Material

vbac032_Supplementary_Data
