# Supplementary material for: NeoSplice: a bioinformatics method for prediction of splice variant neoantigens
Source: Bioinform Adv. 2022 May 6;2(1):vbac032. doi: 10.1093/bioadv/vbac032 (PMC9154024; doi:10.1093/bioadv/vbac032)
Supplement: vbac032_Supplementary_Data [file vbac032_supplementary_data.docx]

**Supplementary methods**

NeoSplice method

*Step 1a. Construction of the BWT data structure and k-mer counting method.*

To efficiently determine the number of occurrences of an arbitrary sequence s in an RNA-seq dataset ***D***, we organize the reads from ***D*** into a suffix array[^1^](https://paperpile.com/c/Tingtu/XQsCX), a sorted array of all suffixes of every read in ***D***. The suffix array enables the number of occurrences of an arbitrary sequence ***T*** to be determined in time *O(|t|)* where *|t|* is the sequence length. The time to create a suffix array is linear in the number of reads.

As a suffix array for an RNA-seq dataset would be prohibitively large, we use the Burrows-Wheeler Transform (BWT) method, which provides the same functionality using a compressed representation of the suffix array. We use the multi-string BWT tool (MSBWT 0.3.0)[^2^](https://paperpile.com/c/Tingtu/ADw8X) to build separate multi-string BWT indexes for the tumor and normal RNA-seq reads. The RNA-seq reads are extracted from aligned BAM files and soft clipped portions of reads are removed. All reads are represented on the same reference strand. The FM index[^3^](https://paperpile.com/c/Tingtu/9Y7Lo) of the BWT consists of counts information for the BWT data structure and rapidly locates the first index and last index of all occurrences of a given sequence (all occurrences appear consecutively in the suffix array). The number of occurrences of the sequence is the difference between the first and last index.

*Step 1b. Tumor specific k-mer searching algorithm.*

A sequence whose occurrence count in the tumor RNA-seq ***T*** exceeds a threshold ***T_min*** (i.e. occurs sufficiently frequently) while its occurrence in the normal RNA-seq ***N*** is less than a maximum count ***N_max*** (i.e. is sufficiently rare) is a considered tumor specific k-mer. Typical values for ***T_min*** are 20-35 and typical values for ***N_max*** are 1-4. Tumor-specific sequences are discovered by a depth-first search process operating in parallel on the tumor and normal BWT data structures.

The search is initialized starting from a 1-mer (e.g. nucleotide "A"). Clearly the sequence "A" will have a huge number of occurrences in both ***T*** and ***N***, so will not satisfy the rarity condition in ***N***. The search is then refined by adding a nucleotide from {A, T, C, G} in front of the current search sequence, then recursively applying the search for the extended k-mer. The recursive search will either yield one or more tumor specific k-mers, or will backtrack from the refinement because the occurrence count of the search k-mer falls below ***T_min***. In this fashion all possible tumor specific k-mers will be found. Maximum k-mer lengths are typically set to 90% of minimum read lengths for ***T*** and ***N****.* The algorithm can be run in a parallelized manner by starting a separate search from each different 1-mer (4 threads) or 2-mer (16 threads) or 3-mer (64 threads), etc. A sublinear speedup was observed when testing on typical Illumina fastq files using 1-thread, 4-threads, and 16-threads of parallelism.

Tumor-specific k-mers of variable length are returned after the search terminates. An Aho–Corasick algorithm (pyahocorasick 1.4.0)[^4^](https://paperpile.com/c/Tingtu/LOARA) is then used to search for the reads that contain tumor specific k-mers in the tumor RNA-seq BAM file. This method runs in time linear with primarily the size of the BAM file. For each occurrence, the k-mer-containing portion of the read along with corresponding quality scores and Cigar strings is written to a new BAM file.

*Step 2a. Splice graph construction.*

The splice graph is a weighted, directed graph. Nodes in the splice graph represent genomic coordinates. Edges in the splice graph represent transcribed intervals (exons) or splices. In addition, splice graphs may carry additional information about insertions, deletions, and single nucleotide variants (SNV), as well as annotations like translation start sites for coding regions. The splice graph is constructed using an RNA-seq BAM file (typically aligned using STAR 2-pass) and GENCODE GFF3 annotation file[^5^](https://paperpile.com/c/Tingtu/Ngo4C). The splice junction, insertion, and deletion information are detected from CIGAR strings of reads in the BAM file using pysam 0.14.153[^6^](https://paperpile.com/c/Tingtu/aiQkU). The exon and SNV information are retrieved by examining aligned reads at each genomic coordinate using pysam 0.14.153. Specifically, for each genomic position with RNA-seq read coverage, different nucleotides are counted by the number of supporting reads. The nucleotide that is different from the reference nucleotide and having the highest number of supporting reads compared with other non-reference nucleotides is selected as the potential SNV. If the p-value of a one sided binomial test (the null hypothesis sequencing error probability for a base by default set to 0.0026, which is the average substitution error rate for Illumina HiSeq R1 reads[^7^](https://paperpile.com/c/Tingtu/sKk2)) at this position is smaller than a user specified threshold and the number of reads supporting the SNV is greater than or equal to a user specified minimum threshold (typically set to 10 – this minimum read threshold also applies to insertions and deletions), a SNV edge for SNV nucleotide will be constructed in the splice graph with weight equal to the number of reads supporting the SNV nucleotide. After the SNV edge is added, a corresponding one nucleotide exon edge representing the reference nucleotide will also be added to the splice graph with weight equal to the number of reads supporting nucleotides different from the SNV nucleotide. If an exon edge covers the start positions or end positions of other types of edges, it will be split into contiguous exon edges (sub-exons) at these positions to allow the transcript path to take exon edges or other types of edges (splice, insertion, deletion, SNV) during splice graph traversal in step 2b. Annotated translation initiation site information is retrieved from the GENCODE GFF3 file[^5^](https://paperpile.com/c/Tingtu/Ngo4C).

*Step 2b. Splice graph traversal algorithm.*

Tumor specific splice junctions are identified by taking the intersection of splice junctions identified within tumor specific k-mers (step 1) and splice junctions found by RNA-seq quantification (step 2a) that are found to be highly expressed in tumor but lowly expressed in normal RNA-seq data (typically set to tumor expression threshold ≥20 and normal expression threshold ≤2). Tumor-specific k-mers that include tumor-specific splice junctions are mapped to the splice graph using CIGAR strings. If any end of the k-mer is mapped inside an exon edge, the k-mer graph path includes the whole exon edge.

For each tumor-specific k-mer graph path supported by a sufficient number of k-mer containing reads (typically set to 10 reads), a depth-first search algorithm is used for graph traversal upstream and downstream of the tumor-specific k-mer graph path. The depth-first search is restricted to edges supported by a sufficient number of paired-end reads that contain a tumor-specific k-mer. If the depth-first search did not reach an annotated transcript's start codon, reference transcripts that cover the k-mer graph path are used for open reading frame inference. Specifically, if an annotated transcript is supported by a path in the splice graph, graph traversal will start from the annotated start codon, follow the annotated exon and splice junction path, and stop when it reaches the upstream depth first search stop position.

*Step 3. Prediction of splice variant neoantigens.*

The transcript sequence identified by depth-first search is then concatenated with the tumor specific k-mer sequence and translated into 8-11mer peptides for MHC-I neoantigen prediction. Binding affinity to MHC molecules expressed by the tumor for in-silico generated peptides is predicted using NetMHCpan-4.0 (12). To further filter for sequences that may be present in normal cells, a reference peptidome is generated by translating protein coding transcripts present in a GENCODE GFF3 annotation file[^5^](https://paperpile.com/c/Tingtu/Ngo4C). Peptides with an IC_50_ value of less than 500 nM for at least 1 MHC allele and not present in the reference peptidome are considered predicted neoantigens.

**Supplementary figures**

**
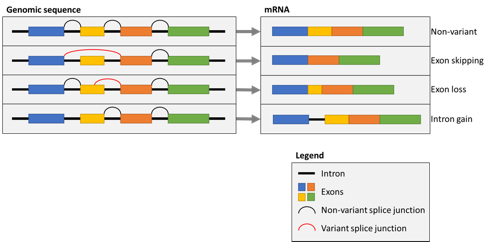
**

***Supplemental Figure 1:* Graphical summary of each type of splice variant (SV) types, including exon skipping, exon loss, and intron gain.** Left represents the splicing pattern of the genomic sequence. Right represents the post-splice mRNA sequence of non-variant as well as each SV type.

*
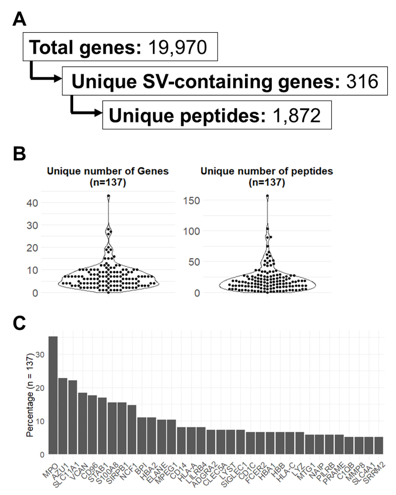
*

***Supplemental Figure 2:* Summary of splice variant (SV) antigens predicted from TCGA LAML dataset.** *(A) Number of total genes, unique SV-containing genes, and unique peptides derived from SV antigens from the TGCA LAML dataset. (B) Summary of number of unique genes (left) and peptides (right) derived from SV antigens per sample within the TCGA LAML dataset. (C) Summary of genes containing predicted SV shared in >5% of TCGA LAML samples.*

**References**

1. [Manber, U. & Myers, G. Suffix Arrays: A New Method for On-Line String Searches. *SIAM Journal on Computing* vol. 22 935–948 (1993).](http://paperpile.com/b/Tingtu/XQsCX)

2. [Holt, J. & McMillan, L. Merging of multi-string BWTs with applications. *Bioinformatics* **30**, 3524–3531 (2014).](http://paperpile.com/b/Tingtu/ADw8X)

3. [Ferragina, P. & Manzini, G. Opportunistic data structures with applications. in *Proceedings 41st Annual Symposium on Foundations of Computer Science* 390–398 (2000).](http://paperpile.com/b/Tingtu/9Y7Lo)

4. [Aho, A. V. & Corasick, M. J. Efficient string matching: an aid to bibliographic search. *Commun. ACM* **18**, 333–340 (1975).](http://paperpile.com/b/Tingtu/LOARA)

5. [Frankish, A. *et al.* GENCODE reference annotation for the human and mouse genomes. *Nucleic Acids Res.* **47**, D766–D773 (2019).](http://paperpile.com/b/Tingtu/Ngo4C)

6. [Heger, A., Belgrad, T. G., Goodson, M. & Jacobs, K. pysam: Python interface for the SAM. *BAM sequence alignment and mapping format* (2014).](http://paperpile.com/b/Tingtu/aiQkU)

7. [Schirmer, M., D’Amore, R., Ijaz, U. Z., Hall, N. & Quince, C. Illumina error profiles: resolving fine-scale variation in metagenomic sequencing data. *BMC Bioinformatics* **17**, 125 (2016).](http://paperpile.com/b/Tingtu/sKk2)
